# Anifrolumab has a direct immunoregulatory effect on inflamed keratinocytes: implications for the treatment of lupus erythematosus skin lesions

**DOI:** 10.3389/fimmu.2025.1648001

**Published:** 2025-10-13

**Authors:** Ksenia Kalyniuk, Tanja Fetter, Marie Grützbach, Tugce Guel, Natalija Novak, Joerg Wenzel

**Affiliations:** Department for Dermatology and Allergy, Center for Skin Diseases, University Hospital Bonn, Bonn, Germany

**Keywords:** lupus, SLE, skin, interferon, keratinocyte

## Abstract

Cutaneous lupus erythematosus (CLE) is an autoimmune skin disease characterized by a type I interferon (IFN)-driven interface dermatitis in which cytotoxic lymphocytes invade the basal layer of the epidermis and induce the keratinocytic cell death. Anifrolumab is a monoclonal antibody targeting the type I interferon receptor (IFNAR1) approved for the therapy of systemic lupus erythematosus (SLE). Recent clinical observations indicated that anifrolumab might be particularly effective in the treatment of lupus erythematosus (LE) skin manifestations. We hypothesize that anifrolumab does not only inhibit interferons circulating in the blood but also has a direct impact on keratinocytes. Our results show that IFNAR1 is expressed in lesional keratinocytes in CLE patients in immunohistochemistry. Gene expression analyses confirmed a strong activation of the interferon signaling pathway in CLE lesions. *In vitro* experiments with HaCaT cells, N/TERT cells and normal epidermal human keratinocyte 3D-epidermis models demonstrated that anifrolumab inhibits the expression of CLE-typical IFN-mediated proteins, including MxA and CXCL10 expression after stimulation with IFNα and synthetic and endogenous immunogenic nucleic acids. This study demonstrates that anifrolumab not only suppresses the type I IFN effect, but also inhibits other pathways of keratinocyte stimulation including pattern recognition receptor (PRR)-activation and chemokine signaling pathways, which are crucial player in the autoamplification of the proinflammatory vicious circle in CLE. These results suggest that the direct effect of anifrolumab on keratinocytes may be an important factor in its clinical efficacy in LE skin lesions and may explain the beneficial clinical effects of anifrolumab specifically in LE skin lesions.

## Introduction

Cutaneous lupus erythematosus (CLE) is an autoimmune skin disorder, characterized by an interferon-associated interface dermatitis (1). The interface dermatitis presents with an anti-epithelial immune response in which cytotoxic lymphocytes penetrate into the basal layer of epidermis and cause keratinocytic cell death (2). This results in the lesional release of cell debris, in which RNA and DNA fragments can have an immunogenic effect and stimulate the innate immune system via TLR-dependent and TLR-independent mechanisms (3). Interferon-associated proinflammatory cytokines, in particular CXCL10 (C-X-C motif chemokine ligand 10), are the main driving force of this lesional vicious circle. These cytokines are expressed in exactly those areas of interface dermatitis in CLE where effector lymphocytes, expressing the corresponding receptor CXCR3, invade the epidermis and trigger keratinocytic cell death (4). The most common clinical subtypes of CLE are chronic discoid LE (CDLE), which presents with scarring skin lesions, subacute cutaneous LE (SCLE), which is characterized by anular/psoriasiform lesions and acute cutaneous LE (ACLE) which presents with acute lesions as well as malar rash and is closely associated to systemic LE (SLE) (1).

In recent years, various strategies have been established to therapeutically interrupt the resulting vicious circle of chronic inflammation. Antimalarials are the standard-of-care-treatment in CLE according to current skin lupus guidelines (1). These drugs bind circulating extracellular immunostimulatory nucleic acids and thus inhibit the endosomal stimulation of pattern-recognition receptors of the innate immune system (5). Corticosteroids and other immunosuppressants, such as methotrexate, are used in treatment-resistant cases, but their use is often limited by side effects. In recent years two biologic drugs, belimumab and anifrolumab, have been approved for the treatment of systemic LE (SLE) and these drugs have shown some efficacy in skin lesions in SLE-patients and also in primary CLE (1).

**Figure 1:**
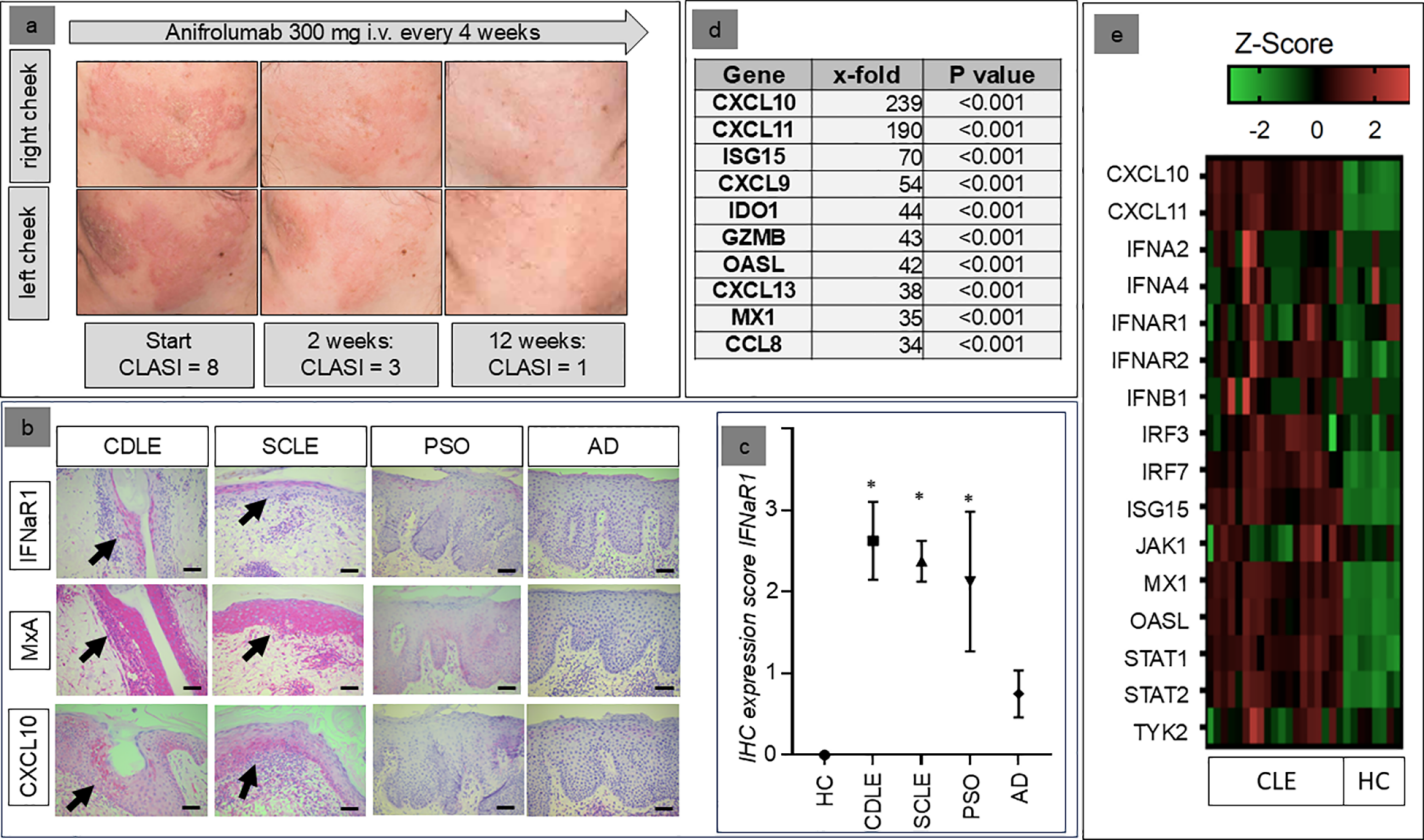
**(a)** Clinical example of the response of the skin lesions of a 36-year old female patient to anifrolumab, resistant to earlier systemic treatment with mycophenolate mofetil (2g/d) and methotrexate (15mg/week). **(b)** Examples of the expression patterns of IFNAR1, MxA, and CXCL10 in healthy individuals (HC=healthy control) and patients with different inflammatory skin disorders (AD, atopic dermatitis; PSO, psoriasis; CDLE, chronic discoid lupus erythematosus; SCLE, subacute cutaneous lupus erythematosus; immunohistochemical staining in red, original magnification: x200, arrows indicate areas with most extensive expression, scale bar = 0.1 mm). **(c)** Mean immunohistochemical staining expression of the IFNαβ-receptor (IFNAR1) in patients with chronic discoid lupus erythematosus (CDLE), subacute cutaneous lupus erythematosus (SCLE), atopic dermatitis (AD) and psoriasis (PSO) compared to healthy controls (HC) (+/- standard deviation, *p<0.05, Kruskal–Wallis test, followed by Dunn’s *post hoc* test). **(d)** Top 10 upregulated genes within CLE skin lesions compared to healthy skin (x-fold expression, Welch’s t-test). **(e)** Individual expression of genes within the Reactome™ pathway “IFNαβ-signaling” in the skin of CLE patients and healthy controls.

In our department we observed the case of a 36-year-old female SLE patient with facial skin lesions, recalcitrant to hydroxychloroquine, mycophenolate mofetil and methotrexate, which responded very quickly to anifrolumab (**Figure 1A**). Anifrolumab is a monoclonal antibody that specifically binds to the type I interferon receptor subunit 1 (IFNAR1), thereby blocking the signaling of type I interferons (IFN) (6). This mechanism of action has effects on various cell types and signaling pathways involved in the pathogenesis of both SLE and CLE. This is particularly well documented for classical immune cells, including lymphocytes, plasmacytoid dendritic cells and monocytes (6–8). The drug might also mediate effects via keratinocytes but these mechanisms have not yet been identified (1). The aim of this study therefore was to investigate the direct effect of anifrolumab on these cells, as this would help to better understand the drug’s efficacy particularly for treating LE skin lesions.

## Patients and methods

### Skin samples and gene expression profiles

All punch biopsies of patients with different inflammatory skin disorders (N = 20) were taken for diagnostic purposes from active skin lesions (CDLE, n=5; SCLE, n=5; atopic dermatitis, n=5; psoriasis, n=5). Biopsies of safety margin skin tumor surgery remnants were used as healthy controls (n=5). Skin samples were fixed with 4% formalin overnight and proceeded for histology and immunohistochemistry. The study was performed in accordance to the principles of the Declaration of Helsinki and approved by the local ethics committee in Bonn (BN 09004). The patients provided written informed consent to participate in this study.

In addition, we analyzed data from Gene Expression Omnibus (GEO GSE280220) including gene-expression profiles of 19 lesional CLE skin biopsies (CDLE and SCLE) and 8 controls (healthy skin) (9).

### Histology & immunohistochemistry

The biopsy samples were subjected to hematoxylin and eosin (H&E) staining to facilitate diagnostic evaluation, which was conducted by a board-certified dermatopathologist (JW). The three-dimensional epidermis models (MatTek) were stained with H&E as well. Immunohistochemistry was conducted with DAKO-Omnis using the Envision staining system (Agilent, Glostrup, Denmark). The staining intensity was assessed on a scale (0=zero, 1=low, 2=moderate, 3=strong) as described before (10). For the evaluation of these staining results, Group differences were assessed using the nonparametric Kruskal–Wallis test, followed by Dunn’s *post hoc* test with correction for multiple comparisons. One-tailed p-values < 0.05 were considered statistically significant. All analyses were performed using GraphPad Prism (version 9.5). The specific antibodies used were those directed towards IFNAR1 (AA 28-227, antikoerper-online.de), CXCL10 (Abcam, ab9807, Cambridge, United Kingdom), and MxA (M143, Prof. Haller, Freiburg).

### Cell culture experiments

*In vitro* experiments utilized immortalized keratinocytes HaCaT (CLS Cell Line Service GmbH, Eppelheim, Germany), N/TERT (provided by collaborators at Biomedical Center II, Bonn), and a 3D epidermis model, constructed from normal epidermal human keratinocytes (=NHEK: EPI-200/EPI-212) from MatTek Life Sciences Biotechnology Company, Bratislava, Slovak Republic. Cell cultivation followed the protocols provided by the respective companies. Cultivation conditions were maintained at 36.6°C, 5% CO_2_ concentration in the air, 95% relative humidity, and 21% oxygen content.

For the HaCaT cell line, DMEM medium supplemented with 10% FCS and 5% PBS was used. N/TERT keratinocytes were cultured in Keratinocyte-SFM medium, which contained all necessary supplements. Cells were split at 80% confluence using Trypsin-EDTA for HaCaT cells and Accutase or N/TERT cells, following the manufacturer’s protocol (all reagents purchased from Thermo Fisher, Waltham, USA).

During the experiments, cells were first treated with the IFN-α receptor blocker anifrolumab (AstraZeneca, Cambridge, UK) at a target concentration of 10 µg/ml and then incubated for 1 h. Subsequently, the cells were treated with various stimuli: 1µg/ml IFN-α (PeproTech, Hamburg, Germany), 10 µg/ml PolyIC (InvivoGen, San Diego, USA), 1 µg/ml PolydAdT (InvivoGen, San Diego, USA), and 12.5 µg/ml eNA extracted from the N/TERT cell line using the NucleoSpin-Kit (Macherey-Nagel, Dueren, Germany). For transfection, a volume of 12.5 µl/ml Lipofectamine 2000 (Invitrogen, Carlsbad, USA) was used to ensure intracellular delivery of PolydAdT and eNA. Cells were then incubated for 24 hours at 37°C. All experiments were implemented in biological triplicates.

After 24 hours, an enzyme-linked immunosorbent assay for human CXCL10 was performed using a CXCL10 DuoSet ELISA (DY266, R&D Systems, Minneapolis, USA), following the company’s protocol. Measurements were taken with the Synergy HT Multi-Detection Microplate Reader (BioTek, Winooski, VT, United States) and analyzed using Gen5 software (Version 1.11.5).

### Next generation sequencing and statistical analyses

RNA was processed by the Next Generation Sequencing (NGS) Core Facility of the Medical Faculty of the University of Bonn using the QuantSeq 3’-mRNA Library Prep Kit by Lexogen. Illumina HiSeq 2500 was used for RNA sequencing (Standard 3’RNA seq with 50 cycles). NGS-gene expression was analyzed with Subio™ using Welch’s t-test. Statistical analysis of ELISA-analyses were performed with GraphPad prism software (version 9.5) using one-sided Kruskal-Wallis test and Mann-Whitney U-test. Analysis of GEO-based gene-expression data was performed using the nSolver platform from Nanostring/Bruker™ and Welch’s t-test (https://nanostring.app.box.com). Confidence intervals were determined at 95%. P < 0.05 was considered as “significant” (*), p < 0.01 as “highly significant” (**), p < 0.1 as “tendency (TD)”. KEGG and Reactome pathways were mapped to differentially expressed genes using DAVID v2024q2 (Database for Annotation, Visualization and Integrated Discovery) and EnrichR (https://maayanlab.cloud/Enrichr) based on Fisher’s exact test. Z-score was calculated using the following formula: (X=data point - μ=mean of the data set)/σ=standard deviation of the data set.

## Results

### Lesional keratinocytes in CLE skin lesions strongly express the IFNαβ-receptor R1

In the first step, immunohistochemistry was used to investigate the extent to which IFNaR1, the target of anifrolumab, is expressed by lesional keratinocytes in patients with CLE. Our analyses showed that both in CDLE and SCLE this receptor is not only expressed by infiltrating cells, but also strongly by epidermal keratinocytes. This mode of expression was significantly stronger than in patients with atopic dermatitis and healthy controls, while a moderate expression was also found in patients with psoriasis. High expression of the IFNaR1 was closely associated with a stronger lesional expression of the IFN-inducible proteins MxA and CXCL10 (**Figures 1B, C**).

### Lesional IFN-signature in CLE includes upregulation of the IFNαβ signaling pathway

In parallel we analyzed gene expression profiles of lesional CLE skin biopsies focusing on the IFN-pathway. The analyses revealed a strong activation of the IFN-associated signaling pathways. Importantly, these analyses supported our immunohistological data with CXCL10 (239-fold) and MxA (also known as Mx1: 35-fold) being strongly upregulated in CLE versus healthy control (**Figure 1D**). The top 10 most upregulated genes also included other ligands of CXCR3 (CXCL9 and CXCL11) and other proinflammatory cytokines (CXCL13, CCL8) as well as typical IFN-regulated genes (ISG15, IDO1, OASL) and GZMB, an IFN-regulated cytotoxic marker. **Figure 1E** details the expression of typical IFN-associated markers, including upregulation of the IFNαβ-receptors in individual patients. In reactome analyses “Interferon-αβ-Signaling” was among the top 20 activated pathways. These analyses reflected the strong activation of immune pathways of the innate (“Innate Immune System”, Cytokine Signaling”, “Toll-like Receptor Cascades”, “Interferon Signaling”, “Interleukin-1 Family Signaling “, “Neutrophil Degranulation”) and the adaptive immune system (“Adaptive Immune System”, “Immunoregulatory Interactions Between a Lymphoid and a non-Lymphoid Cell”) in parallel (**Table 1**).

**Table 1:** Top 20 Reactome pathways in CLE versus healthy control compared to Anifrolumab-effect on eNA-stimulated N/TERT-keratinocytes.

Term	CLE vs HC			Anifrolumab effect		
	Overlap	P-value		Overlap	P-value	
**Immune System**	289/2150	1.26E-171	**	148/2150	2.96E+08	**
**Cytokine Signaling in Immune System**	164/776	4.55E-109	**	93/776	1.68E-01	**
**Signaling by Interleukins**	114/452	1.89E-77	**	44/452	2.51E+09	**
Innate Immune System	129/1149	6.80E-46	**	60/1149	0.169	ns
Adaptive Immune System	110/854	2.57E-41	**	39/854	0.546	ns
**Interleukin-10 Signaling**	29/46	4.81E-24	**	17/46	2.12E+12	**
**Chemokine Receptors Bind Chemokines**	31/57	1.03E-21	**	21/57	0.015	*
Immunoregulatory Interactions Between a Lymphoid and a non-Lymphoid Cell	50/223	1.62E-21	**	6/223	0.947	ns
**Interleukin-4 and Interleukin-13 Signaling**	38/112	3.30E-20	**	13/112	0.002	**
**Interferon Gamma Signaling**	33/99	2.84E-15	**	18/99	5.44E+08	**
**Toll-like Receptor Cascades**	40/174	1.33E-14	**	14/174	0.031	*
**Interferon Signaling**	46/280	2.92E-12	**	51/280	2.41E-02	**
**Toll Like Receptor 4 (TLR4) Cascade**	34/148	3.81E-11	**	14/148	0.009	**
**Diseases Associated With the TLR Signaling Cascade**	19/35	1.59E-07	**	12/35	0.076	**
**Diseases of Immune System**	19/35	1.59E-07	**	12/35	0.076	**
Interleukin-1 Family Signaling	30/140	2.98E-07	**	10/140	0.112	ns
**Interferon Alpha Beta Signaling**	24/78	4.20E-06	**	36/78	1.32E-11	**
**Neutrophil Degranulation**	49/478	9.89E-06	**	31/478	0.035	*
**Disease**	110/2131	1.31E-04	**	119/2131	0.014	*
**MyD88 MAL(TIRAP) Cascade Initiated on Plasma Membrane**	26/115	4.51E-05	**	12/115	0.007	**

In comparison, the inhibitory effect of anifrolumab on the corresponding pathways in eNA-stimulated cultured N/TERT-keratinocytes is shown on the right-hand side. (Statistical results of Reactome pathway enrichment analyses: *p<0.05, **p<0.01, ns = not significant, Fisher’s exact test).

### Anifrolumab significantly inhibits the expression of CXCL10 in HaCaT cells after stimulation of immunostimulatory nucleic acids

**Figure 2:**
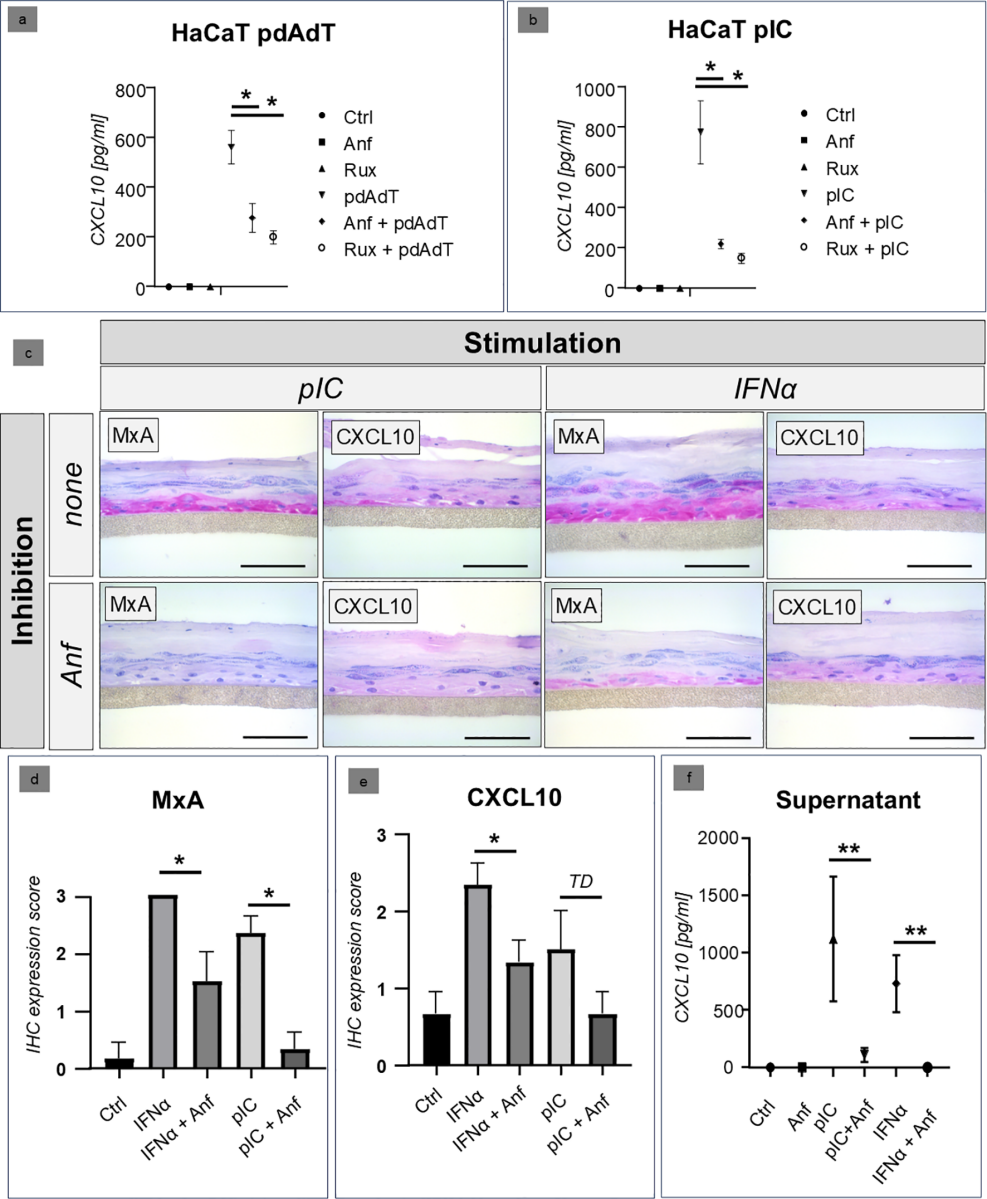
**(a, b)** Comparison of the inhibitory effect of anifrolumab (Anf) with the JAK inhibitor ruxolitinib (Rux) in HaCaT cell culture after stimulation with synthetic RNA (poly I:C/PIC) and DNA (poly(dA:dT)/PdAdT analogues. The figures show the expression of CXCL10 in the supernatant, measured by ELISA (Ctrl = negative control, +/- standard deviation, * p=<0.05, Kruskal-Wallis test). **(c–e)** Inhibitory effect of anifrolumab (Anf) on the lesional type I/III IFN-signature (visualized by MxA and CXCL10 expression using immunohistochemistry, in red, original magnification x400, scale bar = 0.1 mm) in a 3D epidermis model (normal human epidermal keratinocytes), stimulated with poly I:C (PIC) and recombinant IFNα (+/- standard error of mean, *p<0.05, TD = p<0.1, Mann-Whitney U-test). **(f)** Inhibitory effect of anifrolumab (Anf) on the CXCL10 expression in the supernatant of a 3D epidemis model with NHEK cells (normal human epidermal keratinocytes; +/- standard deviation, **p<0.01, Kruskal-Wallis test).

The functional analyses of the efficacy of anifrolumab were initially carried out in a HaCaT cell culture. Stimulation was performed with synthetic immunostimulatory nucleic acids (DNA: polydAdT, RNA: polyIC), which we had already established for an *in vitro* model of CLE. Ruxolitinib, a selective JAK1/2 inhibitor which was established in our *in vitro* system in earlier studies (11), served as a positive control for the inhibition. The studies showed that anifrolumab significantly inhibited the expression of the proinflammatory cytokine CXCL10 after stimulation to a level almost comparable to the inhibitory effect of ruxolitinib (**Figures 2A, B**).

### Confirmation of the efficacy of anifrolumab in a human 3D keratinocyte model

In the next step, we carried out analyses in the 3D keratinocyte model with human cells. Stimulation was performed with the stimulatory RNA polyIC and with recombinant interferon alpha. Our immunohistochemical analyses showed that in this model, anifrolumab was able to significantly reduce both the intraepidermal expression of type I/III-interferon-inducible MxA and the expression of the IFN-regulated proinflammatory cytokine CXCL10 (**Figures 2C–E**). A similar picture was seen in the cell supernatants: Here, treatment with anifrolumab resulted in a highly significant reduction in CXCL10 expression (**Figure 2F**).

### Analyses of the anti-IFNabR effect in N/TERT cells after stimulation with physiological nucleic acids

**Figure 3:**
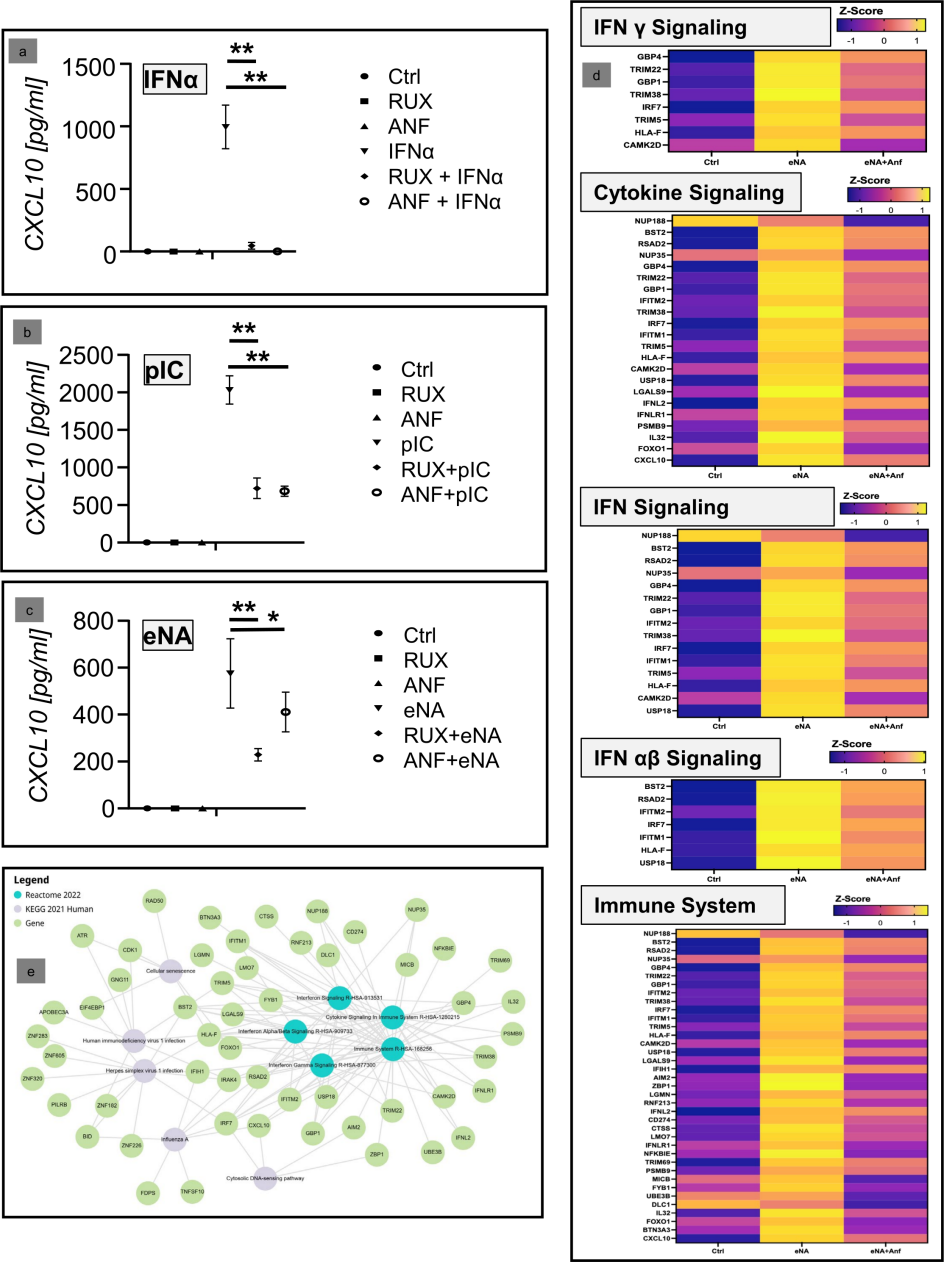
**(a–c)** Inhibitory effect of anifrolumab (Anf) in comparison to ruxolitinib (Rux) in cultured keratinocytes (N/TERT-cell) after stimulation with two synthetic immunostimulatory nucleic acids (PIC; pdAdT) and a physiolological stimulus (extracted nucleic acids, eNA). Depicted is the expression of CXCL10 in the supernatant, measured by ELISA (Ctrl = negative control; +/- standard deviation, *p<0.05, **p<0.01, Kruskal-Wallis test). **(d)** Next generation sequencing analyses of the effect of anifrolumab on the expression of the mRNA of IFN-associated inflammatory pathway molecules. Depicted is the effect of anifrolumab on eNA-stimulated cells in comparison to negative and positive control within the given Reactome pathways as Z-Score. **(e)** Network of the top 5 dysregulated KEGG and Reactome pathways and their associated individual genes affected by the treatment of stimulated N/TERT cells by anifrolumab.

In the final step, the efficacy of anti-IFNaR1 therapy on keratinocytes was confirmed in a further approach. For this purpose, N/TERT cells were stimulated with extracted endogenous nucleic acids, which are an established model for the *in vivo* activation of keratinocytes in CLE skin lesions (12), and then treated with anifrolumab. Stimulation with IFNα and polyIC was carried out in parallel. Here, a significant inhibition of CXCL10 by anifrolumab was shown in all three investigated systems (**Figures 3A–C**).

Finally, next-generation sequencing (NGS) analyses were performed to confirm our earlier results. These analyses showed that anifrolumab significantly inhibits the activation of IFN-associated proinflammatory genes and their pathways in stimulated keratinocytes. In the Reactome analysis, the top regulated pathways (depicted in **Figure 3D**) were “Interferon Signaling” (R-HSA-913531), “Cytokine Signaling in Immune system” (R-HSA-1280215), “Interferon Gamma Signaling” (R-HSA-877300), “Interferon Alpha/Beta Signaling” (R-HSA-909733), and “Immune System” (R-HSA-168256). These pathways include several CLE-typical proinflammatory factors, including IRF7, IFITM1, IFITM2, IFNL2/IL28a and CXCL10, which were downregulated by anifrolumab treatment. Interestingly, when comparing these data with the previously identified top 20 CLE pathways, there was a high degree of consistency in anifrolumab inhibiting these CLE-typical pathways, even though only the effect of the drug on keratinocytes was investigated here (**Table 1**).

Subsequently a network-analysis supported the central role of CXCL10 within the proinflammatory pathways downregulated by anifrolumab (**Figure 3E**).

## Discussion

Anifrolumab is a human monoclonal antibody to the type I IFN receptor subunit 1 (IFNaR1), which blocks the proinflammatory activity of type I IFNs. The drug was first approved for the treatment of moderate to severe systemic lupus erythematosus (SLE) with inadequate response to standard therapies by the US Food and Drug Administration (FDA) in July 2021 and later by the European Medicines Agency (EMA) in February 2022 (13). SLE is an autoimmune disorder involving several organ systems (e.g. kidney, joints, pleura, central nervous system) with a crucial overactivation of both the innate and the adaptive immune system, leading to a strong systemic expression of type I IFNs and pro-inflammatory IFN-regulated cytokines (14). An important cause of chronic inflammation in this disease is a vicious circle that is maintained by continuous reactivation of the innate immune system by factors of the adaptive immune system, in particular immune complexes and pro-inflammatory nucleic acids released as part of a cytotoxic immune response (3, 15). The IFN system is in a central position between these two arms of the immune system and is therefore an ideal target for therapeutic intervention. The efficacy of anifrolumab in SLE has been demonstrated in three clinical trials (MUSE, TULIP-1 TULIP-2), in which, in addition to a significant decrease of SLE disease activity, a reduction of the interferon signature and a beneficial effect on lupus skin lesions was reported (16–20). Anifrolumab downregulates *in vivo* multiple IFN-regulated pathways and has a regulatory effect on apoptosis, innate cell activating chemokines, proinflammatory cytokines and B-cell activation (7). Anifrolumab inhibits the activation of pDCs and thereby reduces the production of type I IFN. It also suppresses the upregulation of costimulatory molecules on stimulated pDCs including CD80 and CD83. The blockade of IFNAR1 also suppresses the differentiation of plasma cells in pDC/B-cell co-cultures and thus reduces the ability of pDCs to stimulate adaptive immune responses (6). pDCs have been described as important type I IFN producers in SLE, but recent studies also highlighted the role of keratinocytes as amplifiers of immune responses in this disease (21, 22). This is specifically relevant for cutaneous LE lesions, in which keratinocytes produce Type I/III IFNs and pro-inflammatory IFN-regulated cytokines. Of the latter, CXCL10 is an important driver of the CLE-typical interface dermatitis (1, 12, 23). Our results demonstrate that anifrolumab has a direct effect on keratinocytes, and in particular significantly downregulates the keratinocytic expression of CXCL10: This analysis was initially conducted in HaCaT cells, a human immortalized keratinocyte cell line derived from healthy human skin, which represents a reliable *in vitro* model for analyzing the inflammatory responses of human keratinocytes (24). Since HaCaT cells may differ from NHEK cells, particularly with regard to the expression of envelope-associated proteins (25), these results were confirmed in two additional keratinocyte systems, N/TERT cells (hTERT-immortalized keratinocytes that can undergo normal differentiation) (26) and a 3D epidermis model derived from NHEK cells, both of which showed the same response to inhibition with anifrolumab. Interestingly, this applies not only to the direct type I IFN effect, but also to other pathways of keratinocyte stimulation: As expected, anifrolumab very effectively inhibits the expression of proinflammatory cytokines after stimulation with recombinant IFNα. However, the drug is also very effective in suppressing these cytokines after stimulation with synthetic and endogenous immunostimulatory nucleic acid fragments, which very likely play an important role *in vivo* in the autoamplification of the immunologic vicious cycle in LE skin lesions (1, 12). Our data further show a direct effect of Anifrolumab on CLE-typical proinflammatory pathways in keratinocytes, by inhibiting “interferon alpha beta signaling” in particular, but also “cytokine signaling in the immune system” and typical PRP pathways such as “toll-like receptor cascades”. This data is supported by earlier case reports, showing improvement of CLE skin lesions, mostly in patients with additional SLE (8, 27, 28), including five cases suffering from discoid LE, one case with chilblain LE and one subacute cutaneous LE (8). In addition, Flouda et al. recently reported a case series of 18 SLE patients with multi-refractory skin disease with a mean CLASI-A (Cutaneous Lupus Erythematosus Disease Area and Severity Index) activity score of 13.9 (13). After a mean of 8.5 months, the skin lesions improved significantly to mean CLASI-A of 3.4 points, with 16 of these 18 patients showing a reduction of the CLASI-A score of ≥ 50% (13). Unfortunately, due to the approval situation of anifrolumab, there are currently only few data on the efficacy of the drug in patients with exclusively cutaneous lupus erythematosus but this topic currently is investigated in a clinical phase III study (NCT06015737). Since Anifrolumab is a human IgG1 antibody and keratinocytes express IFNAR1, there is a theoretical possibility of complement-mediated cytotoxicity via the classical complement cascade. However, keratinocytes are protected by membrane-bound complement regulators such as CD46, CD55, and CD59, and preclinical and clinical data to date do not indicate significant keratinocyte toxicity (29, 30).

In conclusion, our results demonstrate a direct immunoregulatory effect of anifrolumab on stimulated keratinocytes. The fact that these cells express the corresponding receptor IFNaR1 in active LE skin lesions, makes the keratinocytes a very probable target in the treatment of CLE with this drug. This observation could be crucial for a better understanding of the beneficial clinical efficacy of anifrolumab in LE skin lesions.

## Data Availability

The datasets presented in this study can be found in online repositories. The names of the repository/repositories and accession number(s) can be found in the article/supplementary material.
